# The Effects of Different Garlic-Derived Allyl Sulfides on Anaerobic Sulfur Metabolism in the Mouse Kidney

**DOI:** 10.3390/antiox5040046

**Published:** 2016-12-05

**Authors:** Małgorzata Iciek, Anna Bilska-Wilkosz, Magdalena Górny, Maria Sokołowska-Jeżewicz, Danuta Kowalczyk-Pachel

**Affiliations:** Chair of Medical Biochemistry, Medical College, Jagiellonian University, Kopernika 7, Kraków 31-034, Poland; mbbilska@cyf-kr.edu.pl (A.B.-W.); mbgorny@cyf-kr.edu.pl (M.G.); marsokol@wp.pl (M.S.-J.); dkowalczyk@cm-uj.krakow.pl (D.K.-P.)

**Keywords:** sulfane sulfur, hydrogen sulfide, bound sulfur, allyl sulfides, aldehyde dehydrogenase, kidney

## Abstract

Diallyl sulfide (DAS), diallyl disulfide (DADS) and diallyl trisulfide (DATS) are major oil-soluble organosulfur compounds of garlic responsible for most of its pharmacological effects. The present study investigated the influence of repeated *intraperitoneally (ip)* administration of DAS, DADS and DATS on the total level of sulfane sulfur, bound sulfur (S-sulfhydration) and hydrogen sulfide (H_2_S) and on the activity of enzymes, which catalyze sulfane sulfur formation and transfer from a donor to an acceptor in the normal mouse kidney, i.e., γ-cystathionase (CSE) and rhodanese (TST). The activity of aldehyde dehydrogenase (ALDH), which is a redox-sensitive protein, containing an –SH group in its catalytic center, was also determined. The obtained results indicated that all tested compounds significantly increased the activity of TST. Moreover, DADS and DATS increased the total sulfane sulfur level and CSE activity in the normal mouse kidney. ALDH activity was inhibited in the kidney after DATS administration. The results indicated also that none of the studied allyl sulfides affected the level of bound sulfur or H_2_S. Thus, it can be concluded that garlic-derived DADS and DATS can be a source of sulfane sulfur for renal cells but they are not connected with persulfide formation.

## 1. Introduction

Garlic (*Allium sativum*) is native to central Asia and belongs to the Amaryllis family (*Amaryllidaceae*). For thousands of years this plant has been used both for food flavoring and in traditional medicine. Numerous contemporary studies indicate that cysteine-derived organosulfur compounds present in garlic are responsible for most of its pharmacological effects [1,2,3,4,5]. It is also known that some garlic-derived organosulfur compounds contain the so-called sulfane sulfur in their structure. It is a form of sulfur with six valence electrons and no charge (represented by S^0^) which is always attached to another sulfur atom [6]. Sulfane sulfur–containing compounds are present in mammalian cells. They are endogenous metabolites formed from cysteine and/or homocysteine in the processes catalyzed mainly by γ-cystathionase (CSE) and 3-mercaptopyruvate sulfurtransferase (MST) [6,7], while rhodanese (TST) is a sulfane sulfur carrier protein. The Enzyme Commission (EC) numbers of these enzymes are 4.4.1.1, 2.8.1.2, 2.8.1.1, respectively. The sulfane sulfur carrier proteins include also plasma albumin. 

The sulfane sulfur atom is labile and can easily leave the structure of the compound and can readily react with various acceptors, such as thiols (RSH) or cyanide (CN^−^). Sulfur of the latter acceptor is often called the cyanolysable sulfur [8]. There is also the so-called “bound sulfane sulfur”. This term was introduced by Ogasawara to describe sulfur released as hydrogen sulfide (H_2_S) by reducing agents, such as dithiothreitol [9]. That author coined also the term “acid-labile sulfur” to define sulfane sulfur released from an iron sulfur cluster of enzymes by acidification. It is believed that “bound sulfur” and “acid-labile sulfur” can be a fraction of sulfane sulfur [7].

**Scheme 1:**
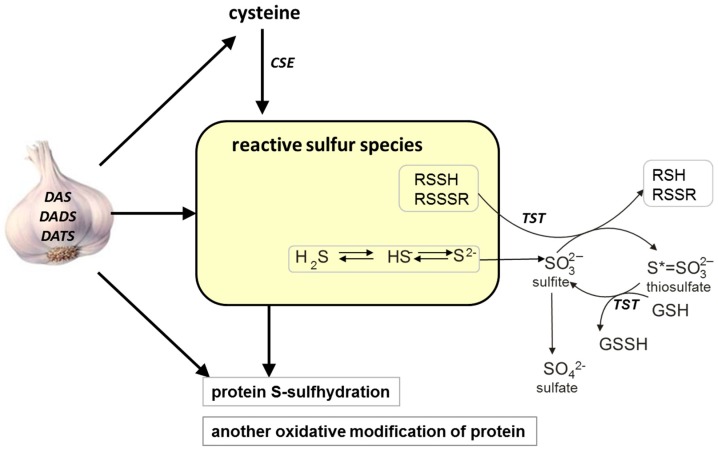
The possible effect of various garlic-derived allyl sulfides, diallyl sulfide (DAS), diallyl disulfide (DADS) and diallyl trisulfide (DATS), on different forms of reactive sulfur species (sulfane sulfur, H_2_S), protein-S-sulfhydration and activity of some redox-sensitive proteins in the mouse kidney. CSE: γ-cystathionase, TSH: rhodanese, GSH: glutathione reduced form, GSSH: glutathione persulfide.

H_2_S was first shown to have a physiological function in 1996 [10]. It is currently known that H_2_S is an important biological messenger molecule that transmits signals by forming persulfide bonds in proteins or low-molecular thiols, where the sulfhydryl group –SH is transformed to an –SSH group, which is referred to as S-sulfhydration (or S-sulfuration) [11,12] (Scheme 1). In recent years, many proteins have been observed to be modified through this process. Especially, as documented by Mustafa et al. [13], liver proteins, including glycerylaldehyde 3-phosphate dehydrogenase (GAPDH), β-tubulin and actin, are sulfhydrated under physiological conditions in the liver homogenate. However, there is very little information about the S-sulfhydration processes in the kidney. It seems to be especially interesting because the concentration of cysteine in the kidney is much higher than in other tissues [14]. Moreover, the physiological level of bound or acid-labile sulfane sulfur is also the highest in the kidney [15]. In addition, an exceptional role of the kidney in sulfur metabolism is corroborated by the fact that the levels of cysteine and products of its metabolism (sulfane sulfur and sulfates) are disturbed in renal insufficiency patients [16,17].

In the present study, the effect of the garlic-derived allyl sulfides, such as diallyl sulfide (DAS), diallyl disulfide (DADS) and diallyl trisulfide (DATS), on the total sulfane sulfur, bound sulfane sulfur and H_2_S, as well as on the activity of CSE and TST, was investigated in the kidney of healthy mice (Scheme 1). The aim of these experiments was to compare the effect of different garlic-derived allyl sulfides on the formation of various forms of reactive sulfur species and on the activity of enzymes participating both in sulfane sulfur formation and transfer from a donor to an acceptor. In addition, we investigated whether the above garlic-derived allyl sulfides can affect the level of bound sulfur in the mouse kidney. Moreover, the activity of aldehyde dehydrogenase (EC 1.2.1.3; ALDH), which is a redox-sensitive protein containing –SH groups in its catalytic center, was studied in the mouse kidney after treatment with various allyl sulfides. We hope that this research will shed a new light on linking the biological activity of garlic-derived allyl sulfides with the formation and transformation of sulfane sulfur in normal kidney cells.

## 2. Materials and Methods

### 2.1. Chemicals

DAS, DADS and DATS were purchased from LKT Laboratories, Inc. (St. Paul, MN, USA) and their purity was >98%. Thiosulfate, formaldehyde, sodium sulfite, ferric chloride and potassium thiocyanate were obtained from the Polish Chemical Reagent Company (P.O.Ch, Gliwice, Poland). All other used chemicals were provided by Sigma Chemical Co. (St. Louis, MO, USA).

### 2.2. Animals and Treatment

The experiment was performed on female albino Swiss mice (weighing approx. 25 g) kept under standard laboratory conditions and fed a standard diet. The animals were randomly divided into groups containing six animals each and were injected *ip* for 10 days with 0.1 mL of oily solution of DAS and DADS (at 350 µm/kg body weight (b.w.)), and DATS at 120 µm/kg b.w. (this compound is very reactive and a dose of 350 µm/kg of b.w. was toxic). The control animals received only the corn oil.

On the 11th day all mice were sacrificed by cervical dislocation. The kidneys were isolated, placed in liquid nitrogen and stored at −76 °C until biochemical assays were performed. The estimated level of reactive sulfur species was expressed in nmoles per g tissue, while the activities of enzymes as μmoles or nmoles of product formed during 1 min per mg protein.

All procedures were approved by the Ethics Committee for the Animal Research in Krakow (number 84/VIII/2007).

### 2.3. Preparation of Tissue Homogenate

The frozen kidneys were weighed and immediately homogenized (1 g of the tissue in 4 mL of 0.1 M phosphate buffer, pH 7.4) using IKA-ULTRA TURRAX T8 homogenizer (IKA Poland Sp. z o.o company, Warsaw, Poland) (6000 rpm for 1 min). Kidney homogenates were kept on ice and used for biochemical assays.

### 2.4. Methods

#### 2.4.1. Total Sulfane Sulfur

This method consists in a nucleophilic attack of cyanide on sulfane sulfur-containing compounds in alkaline solution at a temperature of 10–25 °C [8]. To samples containing 100 μL of homogenate, 80 μL of 1 M NH_3_, 720 μL of distilled water and 100 μL of 0.5 M KCN were added and mixed thoroughly. Then, the mixtures were incubated at room temperature for 45 min and 20 μL of 38% formaldehyde solution and 200 μL of the Goldstein reagent containing Fe^3+^ cation were added. The precipitate formed in this reaction was centrifuged at 10,000× *g* for 10 min and the supernatant was carefully collected. The total sulfane sulfur content was calculated from the absorbance measurement at 460 nm from a standard curve for 1 μM KSCN.

#### 2.4.2. Bound Sulfane Sulfur

This assay was based on the modified method of Ogasawara et al. [18] as described earlier [19]. 

#### 2.4.3. H_2_S Level

The modification of Shen’s method [20] was used for H_2_S level determination. The dominating form of H_2_S at pH higher than 7.0 is S^2−^ ion. This form reacts with zinc acetate yielding zinc sulfide. ZnS reacts with p-phenylenediamine in the presence of FeCl_3_ to form a thionine. Fluorescence was measured with an excitation of 600 nm and an emission of 623 nm.

Aliquots of homogenate (125 μL) were mixed with 125 μL of 1% zinc acetate and 250 μL of 50 μM borate buffer, pH 9.0 followed by incubation at 37 °C for 10 min. Next, the assay was continued according the procedure described for bound sulfane sulfur. H_2_S concentrations were read from a calibration curve prepared from 10 μM Na_2_S.

#### 2.4.4. γ-Cystathionase (CSE) Activity

Enzymatic activity of CSE was determined according to Matsuo and Greenberg [21] with modifications as reported previously [19].

#### 2.4.5. Rhodanese (TST) Activity

The activity of TST was assayed according to Sörbo’s method [22]. Details of this method were defined by Kowalczyk-Pachel et al. [19].

#### 2.4.6. Aldehyde Dehydrogenase (ALDH) Activity

The activity of ALDH was determined by measuring the rate of increase in the absorbance of nicotinamide adenine dinucleotide reduced form (NADH) using propionaldehyde as a substrate as described previously [23]. The rotenone was used to inhibit mitochondrial NADH oxidase and 4-methylpyrazole was added to inhibit alcohol dehydrogenase.

The assay mixture contained 653 μL of sodium phosphate buffer (pH 8.2), 100 μL of kidney homogenate diluted five times with buffer, 200 μL of 1 μM NAD, 10 μL of 1 μM EDTA, 10 μL of 1 μM 4-methylpyrazole and 2 μL of 2 μM rotenone. The reaction was initiated by the addition of 25 μL of 10 μM propionaldehyde as a substrate. The blank sample in which the homogenate was omitted was run simultaneously. The activity of ALDH was calculated using the molar extinction coefficient of NADH of 6.22 mM^−1^·cm^−1^ at 340 nm.

#### 2.4.7. Protein Content

Protein concentration was assayed by the method of Lowry et al. [24] using bovine serum albumin as the standard.

### 2.5. Statistical Analysis

Data are presented as the means ± SD. Statistical significance was determined by a one-way analysis of variance (ANOVA) followed by the Fisher test. Values of *p* < 0.05 were considered significant. The statistical analysis was done using STATISTICA 12.0 Software (Statsoft, Inc., Tulsa, Oklahoma, USA).

## 3. Results

### 3.1. The Effect of Garlic-Derived Allyl Sulfides on Kidney Weight

After the sacrifice of the animals, the kidneys were removed and weighed. The mean kidney weight in each group was calculated and is presented in Table 1. In the groups of animals administered with allyl sulfides a slight increase in the kidney weight was observed in comparison to the control group, but the difference was not statistically significant. The color of the kidney was not affected by the tested compounds.

### 3.2. The Effect of Garlic-Derived Allyl Sulfides (DAS, DADS and DATS) on the Level of Various Reactive Sulfur Species in the Mouse Kidney

**Figure 1:**
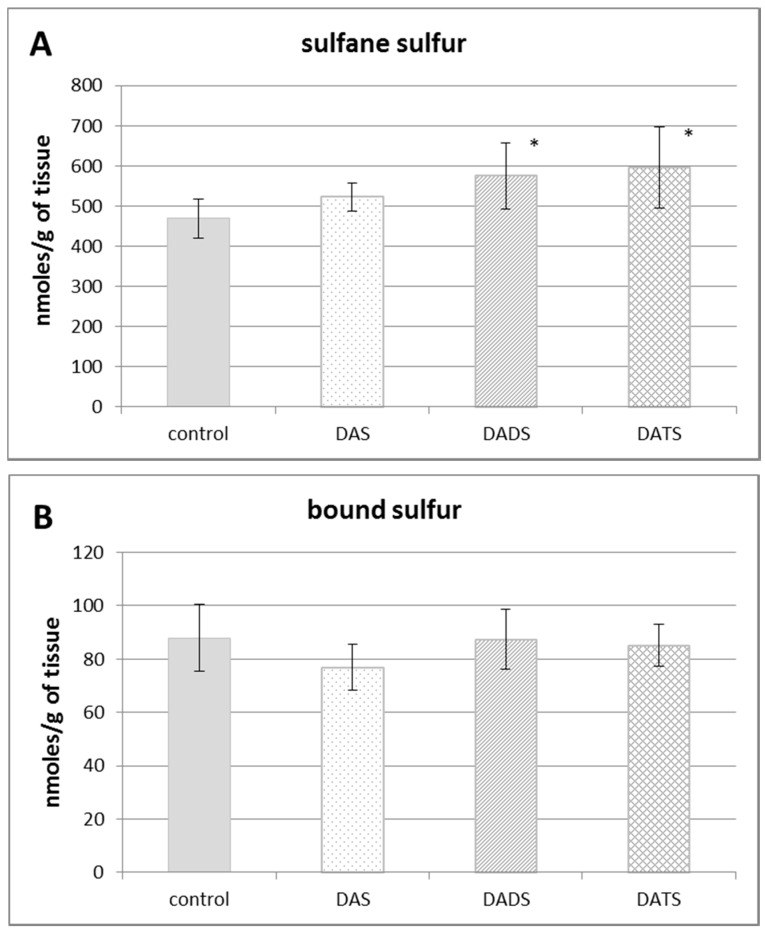

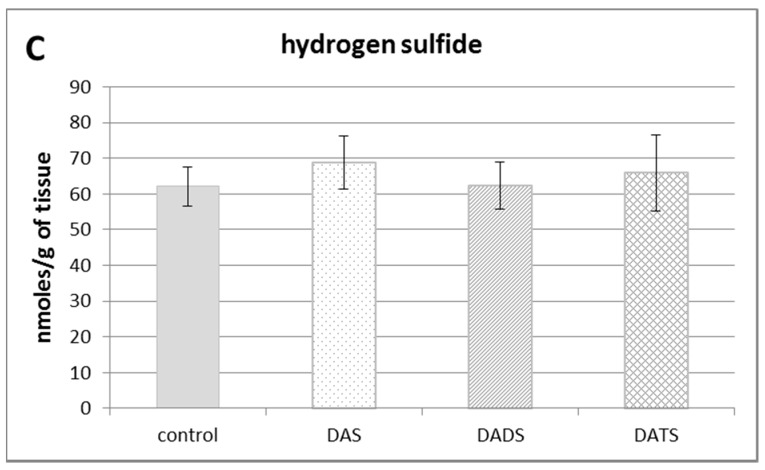
The effect of garlic-derived allyl sulfides (DAS, DADS and DATS) on the level of various reactive sulfur species: total sulfane sulfur (**A**); bound sulfur (**B**) and free hydrogen sulfide; H_2_S (**C**). Data represent the means of six animals in each group ± SD and are expressed as nmoles per 1 g of wet weight. Each assay was performed in triplicate. Statistically significant differences were determined by ANOVA followed by the Fisher test. * Significantly different from control group (*p* < 0.05).

Figure 1 presents the effect of all studied allyl sulfides (DAS, DADS and DATS) on the level of various reactive sulfur species: the total sulfane sulfur, bound sulfur and free H_2_S. The administration of all studied allyl sulfides caused an increase in the total sulfane sulfur, but it was statistically significant only for DADS and DATS (*p* = 0.032 and *p* = 0.013, respectively) (Figure 1A). The highest increase in the concentration of the total sulfane sulfur was observed upon the influence of DATS at the dose three times lower than the concentrations of DADS and DAS.

Figure 1B illustrates the effect of DAS, DADS and DATS on the level of bound sulfur. None of the studied compounds affected the bound sulfur level (mainly hydropersulfides) in the mouse kidney. The level of free H_2_S in the kidney of control animals was approximately 60 nmoles per 1 g of tissue. After administration of allyl sulfides, no statistically significant changes in the free H_2_S level in the mouse kidney were observed (Figure 1C).

### 3.3. The Effect of Garlic-Derived Allyl Sulfides (DAS, DADS and DATS) on the Activities of Sulfurtransferases (CSE and TST) in the Mouse Kidney

**Figure 2:**
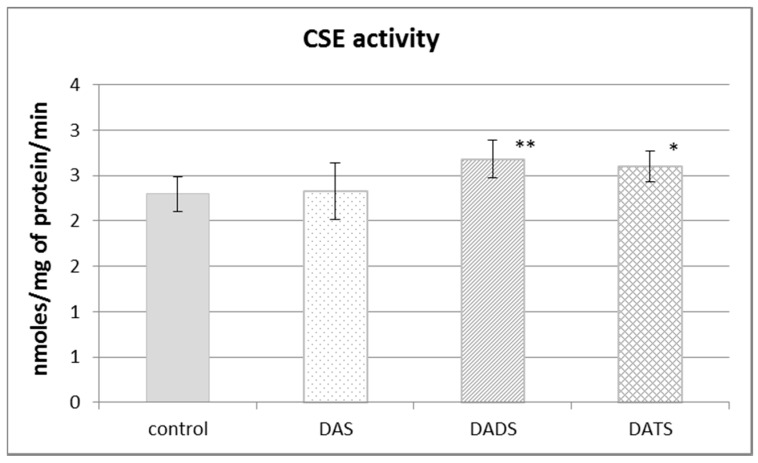
The effect of garlic-derived allyl sulfides (DAS, DADS and DATS) on the activity of γ-cystathionase (CSE). Data represent the means of six animals in each group ± SD and are expressed as nmoles per 1 mg of protein per minute. Each assay was performed in triplicate. Statistically significant differences were determined by ANOVA followed by the Fisher test. * *p* < 0.05, ** *p* < 0.01 significantly different from control group.

**Figure 3:**
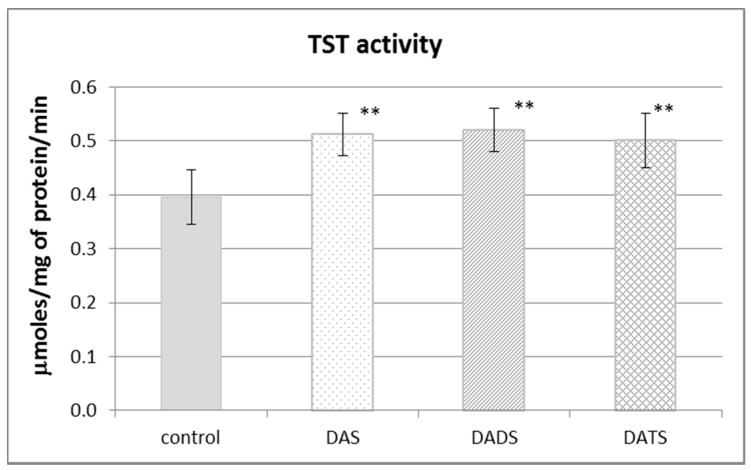
The effect of garlic-derived allyl sulfides (DAS, DADS and DATS) on the activity of rhodanese (TST). Data represent the means of six animals in each group ± SD and are expressed as nmoles per 1 mg of protein per minute. Each assay was performed in triplicate. Statistically significant differences were determined by ANOVA followed by the Fisher test. ** Significantly different from control group (*p* < 0.01).

Figure 2 presents the effect of garlic-derived allyl sulfides on the activity of the CSE, which is the main enzyme involved in the biosynthesis of H_2_S and sulfane sulfur–containing compounds. The present study demonstrated that the activity of CSE was enhanced in the mouse kidney after *ip* administration of DADS and DATS (*p* = 0.009 and *p* = 0.032, respectively) (Figure 2). It means that compounds with sulfane sulfur affect CSE activity, maybe providing the substrate. On the other hand, the activity of TST, which is the enzyme involved mainly in reactive sulfane sulfur transfer, was augmented upon the influence of all studied sulfur compounds: DAS, DADS and DATS (Figure 3).

### 3.4. The Effect of Garlic-Derived Allyl Sulfides (DAS, DADS and DATS) on ALDH Activity

**Figure 4:**
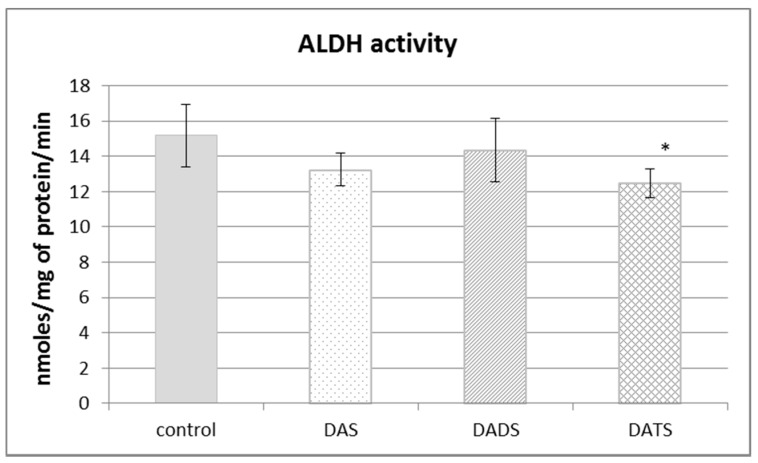
The effect of garlic-derived allyl sulfides (DAS, DADS and DATS) on the activity of aldehyde dehydrogenase (ALDH). Data represent the means of six animals in each group ± SD and are expressed as nmoles per 1 mg of protein per minute. Each assay was performed in triplicate. Statistically significant differences were determined by ANOVA followed by the Fisher test. * Significantly different from control group (*p* < 0.05).

Aldehyde dehydrogenase (ALDH) is not involved in sulfur metabolism but it has a sulfhydryl group in its catalytic center, so its structure and activity can be modified by reactive sulfur species. The obtained results presented in Figure 4 revealed that the ALDH activity was decreased in a significant manner only after DATS treatment (*p* = 0.018).

## 4. Discussion

The present studies demonstrated that DADS and DATS but not DAS significantly increased the total sulfane sulfur level (Figure 1A) and CSE activity (Figure 2) in the mouse kidney cells. These results seem to be logically consistent because CSE catalyzes, inter alia, reactions yielding sulfane sulfur–containing products (thiocysteine, thiocystine) [25,26]. Moreover, Iciek et al. indicated that DADS and DATS elevated the total sulfane sulfur level and the activity of sulfane sulfur biosynthetic enzymes in the mouse liver cells [27]. In addition, apart from the influence on sulfane sulfur and sulfurtransferase activity, DADS increased the number of cells containing glial Gomori-positive granules in the mice [28]. Next, it was demonstrated that this Gomori-positive material contains sulfane sulfur [29].

It is worth noting that DATS is a trisulfide which resembles endogenous thiocystine, whereas DADS in the form of thiosulfoxides is somewhat similar to endogenous thiocysteine. Thus, both DADS and DATS show structural similarity to endogenous metabolites formed during anaerobic cysteine metabolism leading to sulfane sulfur–containing compounds. Hence, it appears that in the present study, DADS and DATS can be both an exogenous source of sulfane sulfur for the kidney and an activator of CSE, which is involved in sulfane sulfur biosynthesis. However, it is known that CSE is responsible also for the endogenous production of H_2_S. Thus, the question arises as to why no increase in the H_2_S level was observed in animals administered DADS and DATS (Figure 1C). It should be remembered that H_2_S is an active molecule and is very toxic in higher concentrations. It means that an imbalance between H_2_S biogenesis, its storage capacity in the hydropersulfide form (–SSH) and biodegradation is dangerous. H_2_S is biodegraded mostly in mitochondria through a series of oxidations catalyzed by the following enzymes: sulfide quinone oxidoreductase (SQR), persulfide dioxygenase (ETHE1), sulfite oxidase (SO) and TST. The H_2_S degradation pathway begins with the reaction catalyzed by SQR. This enzyme catalyzes a two-electron oxidation of H_2_S to the sulfane sulfur–containing persulfide SQR–SSH [30]. The second enzyme participating in H_2_S oxidation ETHE1 catalyzes the production of sulfite which can be oxidized to sulfate by SO or can accept sulfane sulfur from persulfide, yielding thiosulfate in the reaction catalyzed by TST [31]. Therefore, it can be suspected that DADS and DATS do influence H_2_S production in the mouse kidney but it is quickly oxidized to sulfane sulfur compounds. This hypothesis is partially confirmed by the present results, indicating that all three studied allyl sulfides increased the activity of TST (Figure 3), which, as mentioned above, is an enzyme engaged in H_2_S oxidation.

H_2_S formation in the kidney was first described over three decades ago [32]. However, the renal effect of sulfane sulfur and H_2_S has been completely ignored for many years. This situation started to change in the first decade of the third millennium. The decreased level of sulfane sulfur was observed in patients suffering from chronic kidney disease [16]. Wang et al. indicated that ischemia-reperfusion caused a significant decrease in CBS and CSE mRNA and protein levels with a concomitant reduction of glutathione and H_2_S production in the kidney [33]. Moreover, other authors also demonstrated that during many renal diseases, the level of H_2_S production dropped [34]. On the other hand, it is known that some medical conditions are connected with excessive H_2_S production [35,36]. Therefore, it appears that garlic-derived allyl sulfides are characterized by different modes of action in normal and pathological cells. This could explain the reason why none of the allyl sulfides investigated in the present study affected the free H_2_S level in the normal mouse kidney. This is a plausible hypothesis because our previous study showed that DADS increased the concentrations of glutathione and sulfane sulfur and CSE activity in the livers of Ehrlich ascites tumor (EAT)-bearing mice without any changes in the above-mentioned parameters in EAT cells [37].

Earlier studies of Benavides et al. demonstrated non-enzymatic production of H_2_S from DADS and DATS in the presence of GSH in red blood cells [38]. In our study, no increase in the H_2_S level was observed in the kidney after DADS and DATS administration. It could be caused by the collection of the kidneys 24 h after the last dose of the sulfur compounds. Since H_2_S is a sulfur species with a half-life of minutes [39], and it can be expected that a possible increase in the H_2_S level could occur immediately after the administration of allyl sulfides, while at the time when the determination was made, an increase in the sulfane sulfur level was observed. In the aforementioned studies by Benavides et al. [38], the real-time in vitro measurement of the H_2_S level was reported. Moreover, a recent study of DeLeon et al. monitoring the formation of H_2_S and polysulfides from garlic oil in human embryonic kidney cells (HEK 293) revealed that administration of garlic oil to cells increased the intracellular polysulfide (sulfane sulfur) level but had a minimal effect on the H_2_S level [40]. Only after the addition of exogenous thiols (Cys, GSH) to cell culture, an increase in the H_2_S level was observed. It means that the endogenous concentration of thiols could be insufficient to release H_2_S from garlic-derived allyl sulfides. In order to explain in detail the relationship between H_2_S and sulfane sulfur levels after treatment with allyl sulfides, it would be necessary to conduct similar experiments at different times (shorter than 24 h) after the administration of sulfur compounds.

The obtained results also indicated that none of the studied allyl sulfides had an effect on the level of bound sulfane sulfur (Figure 1B). It should be reminded that bound sulfane sulfur is a direct precursor of free H_2_S [9,41]. Thus, considering that none of the studied allyl sulfides had an effect on the level of H_2_S (Figure 1C), these results seem logically consistent.

The present results indicated also that DATS, but not DAS and DADS, decreased ALDH activity in the normal mouse kidney (Figure 4). ALDH catalyzes reactions of aldehyde oxidation to carboxylic acids. Aldehydes are generated from a wide variety of endogenous and exogenous precursors during numerous biochemical processes [42]. More than 200 aldehyde species arise from the oxidative degradation of cellular membrane lipids, including 4-hydroxy-2-nonenal (4-HNE) and malondialdehyde (MDA) [43]. The biologically active ALDH substrate acetaldehyde (ethanal) is formed in the alcohol dehydrogenase (EC 1.1.1.1; ADH)-catalyzed reaction of oxidation of exogenous ethyl alcohol (ethanol or drinking alcohol). Thus, ALDH activity seems essential in terms of both the pathomechanism of many ailments and their therapy.

In turn, although multifarious biological activities of garlic are commonly known and are now under intense examination, the mechanisms of therapeutic action of garlic preparations are still insufficiently understood. We have drawn attention to studies indicating that the mechanism of therapeutic action of garlic-derived compounds is connected with their influence on ALDH activity. Kim et al. indicated that the inhibition of ALDH activity was one of the mechanisms by which DATS suppressed the growth of breast cancer cells in vitro and in vivo [44]. It was observed that ALDH activity in the liver cells was reduced in mice intragastrically administered fresh garlic juice for eight days [45]. Also, our preliminary study indicated that administration of DATS led to the inhibition of ALDH in the normal mouse liver. In summary, in the present study we showed for the first time that DATS inhibited ALDH activity in the mouse kidney. Studies of the effects of garlic-derived compounds on the kidney cells are scarce. The study of Bagheri et al. showed that the garlic juice significantly prevented renal reperfusion-induced functional and histological injuries [46].

## 5. Conclusions

It can be concluded that garlic-derived DADS and DATS influenced the anaerobic cysteine metabolism, increasing the level of sulfane sulfur compounds and elevating the activity of enzymes involved in its formation and transfer in the normal mouse kidney. They did not influence the H_2_S concentration and S-sulfhydration level. However, further experiments are needed in order to get more detailed insight into the mechanism of action of garlic-derived allyl sulfides. Especially, studies of enzymes participating both in the synthesis and biodegradation of H_2_S are required. They should focus not only on the catalytic activity but also on the protein level (antibodies) and/or expression (mRNA) of enzymes engaged in these processes.

The present studies also showed that DATS inhibited ALDH activity in the mouse kidney. It is an interesting observation because it shows a new pharmacological property of garlic-derived allyl trisulfide. ALDH inhibition can lead to an increase in the concentration of aldehydes, the toxicity of which could be used in the elimination of cancer cells.

Taken together, the present results suggest that the mechanism of biological activity of various garlic-derived allyl sulfides may be connected with sulfane sulfur metabolism or with ALDH activity. This issue requires further studies because a better understanding of the mechanism of therapeutic actions of garlic-derived allyl sulfides is important not only for the explanation of their effects but also for their wider clinical use.

## Figures and Tables

**Table 1:** The kidney weight in control and allyl sulfide–treated mice. Data are presented as the means ± SD of six animals in each group.

Group	Kidney Weight (Mean ± SD) (g)
Control	0.277 ± 0.028
DAS	0.307 ± 0.061
DADS	0.308 ± 0.032
DATS	0.289 ± 0.028

DAS: diallyl sulfide, DADS: diallyl disulfide, DATS: diallyl trisulfide.
